# Determination of Free-Form and Peptide Bound Pyrraline in the Commercial Drinks Enriched with Different Protein Hydrolysates

**DOI:** 10.3390/ijms17071053

**Published:** 2016-07-04

**Authors:** Zhili Liang, Lin Li, Haiping Qi, Xia Zhang, Zhenbo Xu, Bing Li

**Affiliations:** 1 School of Food Sciences and Engineering, South China University of Technology, Guangzhou 510640, China; zhililiang1988@gmail.com (Z.L.); oliveauspicious@sina.com (H.Q.); z.xia.scut@gmail.com (X.Z.); zhenbo.xu@hotmail.com (Z.X.); 2 Guangdong Province Key Laboratory for Green Processing of Natural Products and Product Safety, South China University of Technology, Guangzhou 510640, China; 3 University Affairs Committee, Dongguan University of Technology, Dongguan 523808, China; 4 Department of Microbial Pathogenesis, Dental School, University of Maryland, Baltimore, MD 21201, USA

**Keywords:** pyrraline, protein hydrolysates, advanced glycation end products, Maillard reaction, solid-phase extraction

## Abstract

Pyrraline, a causative factor for the recent epidemics of diabetes and cardiovascular disease, is also employed as an indicator to evaluate heat damage and formation of advanced glycation end-products (AGEs) in foods. Peptide-enriched drinks (PEDs) are broadly consumed worldwide due to rapid rate of absorption and perceived health effects. It can be hypothesized that PED is an important source of pyrraline, especially peptide bound pyrraline (Pep-Pyr). In this study we determined free-form pyrraline (Free-Pyr) and Pep-Pyr in drinks enriched with whey protein hydrolysate (WPH), soy protein hydrolysate (SPH) and collagen protein hydrolysate (CPH). A detection method was developed using ultrahigh-performance liquid chromatography with UV-visible detector coupled with tandem mass spectrometry after solid-phase extraction (SPE). The SPE led to excellent recovery rates ranging between 93.2% and 98.5% and a high reproducibility with relative standard deviations (RSD) of <5%. The limits of detection and quantification obtained were 30.4 and 70.3 ng/mL, respectively. Pep-Pyr was identified as the most abundant form (above 96 percent) of total pyrraline, whereas Free-Pyr was present in a small proportion (less than four percent) of total pyrraline. The results indicate that PED is an important extrinsic source of pyrraline, especially Pep-Pyr. As compared with CPH- and SPH-enriched drinks, WPH-enriched drinks contained high content of Pep-Pyr. The Pep-Pyr content is associated with the distribution of peptide lengths and the amino acid compositions of protein in PEDs.

## 1. Introduction

Advanced glycation end products (AGEs) are a heterogeneous group of compounds that attributed to the reaction between reactive aldehydes of reducing saccharides and the free amino groups of proteins. Based on the review of information obtained from animal and human studies concerning the bioavailability and metabolic fate of dietary AGEs [1], Ames has advocated that the dietary AGEs are irrelevant to human health deterioration. However, AGEs are capable of modification on the chemical and biological properties of native molecules by binding to their cellular receptors [2,3], which thus are associated with oxidative stress, inflammation [4], and process that eventually lead to some chronic diseases, e.g., diabetes [5,6,7] and uremia [8,9]. The correlation between AGEs and numerous diabetic [5,6,7] or renal complications [8,9] have also been well documented.

According to the sources, AGEs have been classified into two major classes: endogenous AGEs and exogenous AGEs. Endogenous AGEs are formed in the body under physiologic conditions, which are also called biological-AGEs. Exogenous AGEs, i.e., food-AGEs or dietary AGEs, are produced during the heat processing of food [10]. As the significant contribution of dietary AGEs to the in vivo AGE pool have recently been verified [11,12,13,14,15], dietary AGEs are consequently considered as a class of potential food contaminants. In addition, as the amino group of AGEs mainly derived from amino acids, peptides and proteins, various forms of AGEs were identified based on the structure of amino group, including free-form AGEs, peptide bound AGEs and protein bound AGEs [16,17].

Pyrraline, as an acid-labile AGE, has been found in food as free form [18,19], and peptide and protein bound forms [20,21]. With both close association with shelf life and storage conditions, and stability during prolonged storage time [19,20], pyrraline has been considered to be a suitable AGE for evaluating the content of advanced Maillard reaction products in thermal treated or long stored foods. As low level of free amino acids in food compared with that of peptides and proteins, the peptide bound pyrraline (Pep-Pyr) and protein bound pyrraline may remain the major forms of pyrraline in food processing as compared with the free-form pyrraline (Free-Pyr). In addition, with the minimal bioavailability of Pep-Pyr ranging from 60% to 80% [22], it is noteworthy that only the peptide or protein bound form of pyrraline (especially Pep-Pyr) were found in the circulation in vivo, with an example being the penetration into intestinal cells across the apical membrane by the peptide transporter PEPT1 only observed for Pep-Pyr, but not Free-Pyr. The absorption of dietary pyrraline most likely occurs in the form of dipeptides rather than as the free form, which is not a substrate for the intestinal lysine transporter [23].

Protein hydrolysate is a complex mixture of peptides with different chain lengths and free amino acids, which was obtained by a process of heat-acid treatment or mild proteolytic enzymes incubation [24]. With an application for protein supplementation in energetic drinks, geriatrics products, sports nutrition products and weight control diets [25], protein hydrolysate is an essential ingredient of peptide-enriched drinks (PEDs), and has been widely employed as additives in sport drinks due to the advantage of rapid absorption [24,26]. However, the large amount of di- and tri-peptides contained in protein hydrolysates are absorbed more rapidly than free-form amino acids and intact proteins [27], and some short-chain peptides derived from protein hydrolysates may have some positive bioactivities [28,29]. Thus, the protein hydrolysates are the essential ingredients in functional drinks due to their positive bioactivities. Nevertheless, saccharide (e.g., glucose, fructose and sucrose) is also an essential ingredient of these PEDs, leading to the further production of Free-Pyr and Pep-Pyr by peptides and amino acids reacting with saccharide under thermal treatment in food processing, including pasteurization and ultra-high temperature processing (UHT). Comparing with Free-Pyr in particular, the rapidity of Pep-Pyr formation as aforementioned may lead to the higher potential exposure risk due to high content of short-chain peptides in PEDs.

In previous reports, the pyrraline concentration by various measurement techniques in real food samples have been well studied on the total content of pyrraline, with the differences between Free-Pyr and Pep-Pyr still remaining unclear. However, the extent of risk exposure by dietary pyrraline may be attributed to the different concentrations between Free-Pyr and Pep-Pyr due to their different absorption mechanisms. In addition, with different formation rapidity of Pep-Pyr in PEDs and other food products (mostly containing intact proteins), the individual monitoring on the content of Free-Pyr and Pep-Pyr in PEDs are significantly required.

In the present study, a method was developed and applied to quantify the Free-Pyr and Pep-Pyr in commercial PEDs. Furthermore, the relationship between pyrraline content and the type of protein hydrolysate was investigated. The aim of this work was to evaluate the exposure risk of Free-Pyr and Pep-Pyr when different protein hydrolysates (whey protein hydrolysate (WPH); soy protein hydrolysate (SPH); and collagen protein hydrolysate (CPH)) were applied to commercial drinks.

## 2. Results

**Figure 1:**
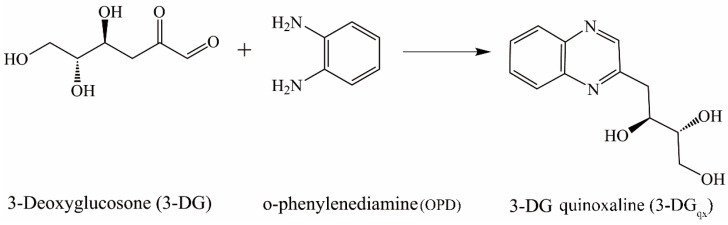
Derivatization of 3-deoxyglucosone (3-DG) by *o*-phenylenediamine (OPD).

The aim of this study was to identify the Free-Pyr and Pep-Pyr in the commercial PEDs and to quantify pyrraline in various PEDs. In order to terminate the Maillard reaction and avoid some lagged reaction effects, 1,2-dicarbonyl compounds in PEDs were converted into their quinoxaline derivatives by *o*-phenylenediamine (OPD) (Figure 1). This method has been successfully applied before to analyze AGEs and 1,2-dicarbonyl compounds in model mixtures and various other matrices [30,31,32,33].

**Figure 2:**
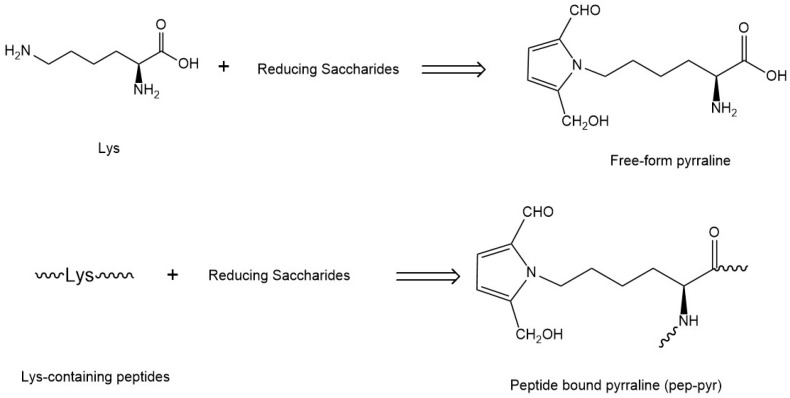
Free form and peptide bound pyrraline formation derived from Lys and peptides, individually.

The amount of Free-Pyr was determined directly after dicarbonyl compounds derivatization followed by solid-phase extraction (SPE) procedure. It was not feasible to quantify Pep-Pyr content due to the diversity of peptides’ structures (Figure 2) and unavailable commercial standard of Pep-Pyr. Therefore, an enzyme hydrolysis procedure was performed after the SPE protocol to release Free-Pyr, the total amount of pyrraline in PED samples was determined by calibration curve with standard pyrraline. The Pep-Pyr concentration was calculated by subtraction of Free-Pyr content from total pyrraline content.

### 2.1. Optimization of Derivatization Procedure

1,2-dicarbonyl compounds, such as 3-deoxyglucosone or 1,4-dideoxyglucosone, are frequently discussed as precursors for AGEs in foods and in vivo. A lagged effect (delayed effect in AGE formation) may occur with the presence of 1,2-dicarbonyl compounds in the samples, consequently leads to unreliable quantification of AGEs. Thus, 1,2-dicarbonyl compounds should be completely deactivated to reduce the lagged effect. 3-deoxyglucosone (3-DG) has proven to be the predominant 1,2-dicarbonyl compound in commonly consumed foods [30]. Accordingly, 3-DG was employed as the marker for 1,2-dicarbonyl compounds deactivation.

**Figure 3:**
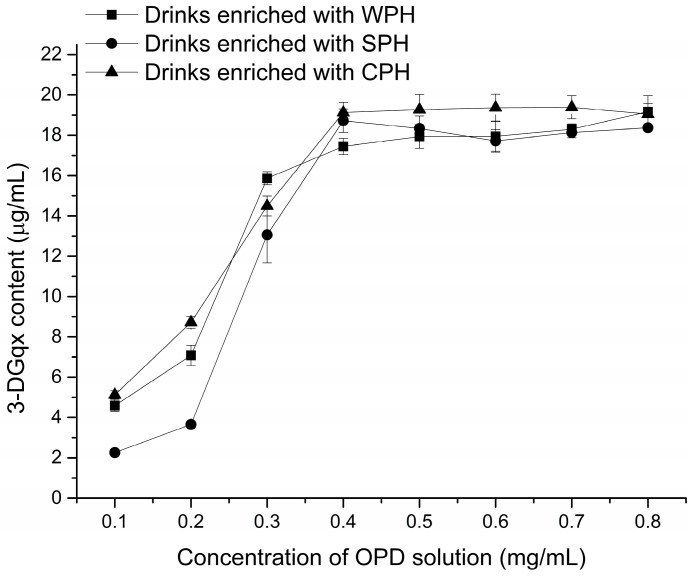
Effect of addition amount of *o*-phenylenediamine (OPD) on measured 3-DG quinoxaline (3-DG_qx_) content in Peptide-enriched drinks (PEDs).

The effect of added OPD on the formation rate of 3-DG quinoxaline (3-DG_qx_) is presented in Figure 3. When the concentration of OPD added was less than 0.4 mg/mL, the content of 3-DG_qx_ increased with increasing amounts of OPD. By contrast, when the concentration of OPD further increased above 0.4 mg/mL, 3-DG_qx_ content reached a plateau, indicating that 3-DG was deactivated completely. Meanwhile, a reliable quantification of 3-DG was finally achieved by addition of derivatization reagent with 0.4 mg/mL OPD (Table 1). Consequently, the added concentration of OPD should be at least 0.4 mg/mL, which can be associated with reducing the lagged effect. There was no significant difference in 3-DG content among different PEDs. Approximately 18 μg/mL of 3-DG was obtained in all commercial PEDs.

### 2.2. Optimization of SPE Procedure

**Figure 4:**
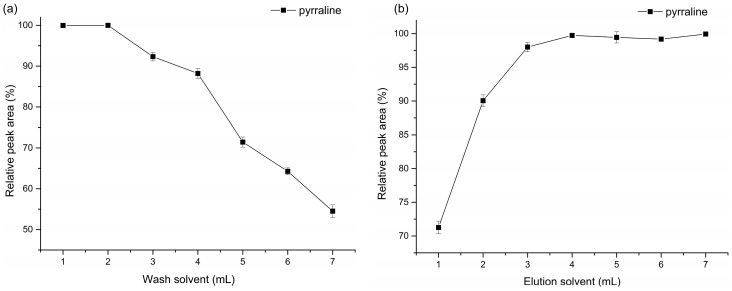
Relationship between volume of washing solvent and recovery of pyrraline (**a**); and relationship between volume of elution solvent and recovery of pyrraline (**b**). (**a**) Volume of elution solvent was 4 mL, the recovery obtained using the least volume of the washing solvent (1 mL) was taken as 100, and the data were expressed as relative peak area; (**b**) Volume of washing solvent was 2 mL, the recovery obtained using the volume of the elution solvent (7 mL) was taken as 100, and the data were expressed as relative peak area

The relationship between volume of washing solvent and recovery of pyrraline is shown in Figure 4a,b. Theoretically, the targeted analytes and the impurities are retained on the sorbent bed when the sample passes through. The impurities are rinsed through with wash solutions that are strong enough to remove them, but weak enough to leave the compounds of interest behind, then the targeted analytes are eluted by elution solvent [34]. Therefore, the volumes of washing and elution solvent are very important to remove interferents and gain an excellent recovery of targeted analytes simultaneously. A balance should be explored between the volume of washing solvent and volume of elution solvent. In Figure 4a, 2 mL was an appropriate volume of washing solvent without pyrraline loss. Figure 4b shows a stable content of pyrraline when the volume of elution solvent was greater than 4 mL. This indicates that elution with 4 mL or more of the acetonitrile can result in complete extraction of pyrraline. As a result, the optimal SPE procedure was obtained with 2 mL of washing solvent and 4 mL of elution solvent.

### 2.3. Purification of PEDs by SPE

**Figure 5:**
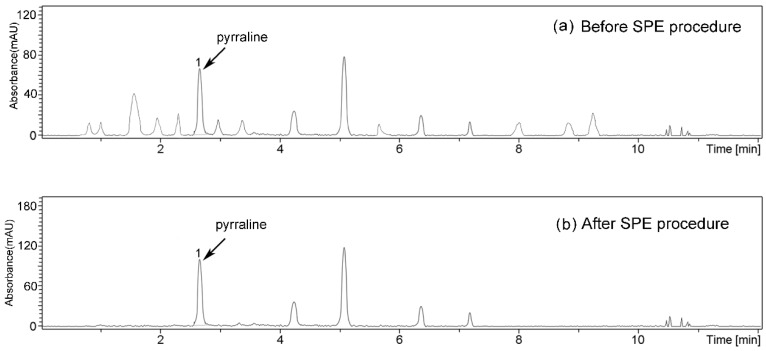
UPLC-UV chromatogram recorded at 297 nm of pyrraline in PEDs: (**a**) before SPE procedure; and (**b**) after SPE procedure.

**Figure 6:**
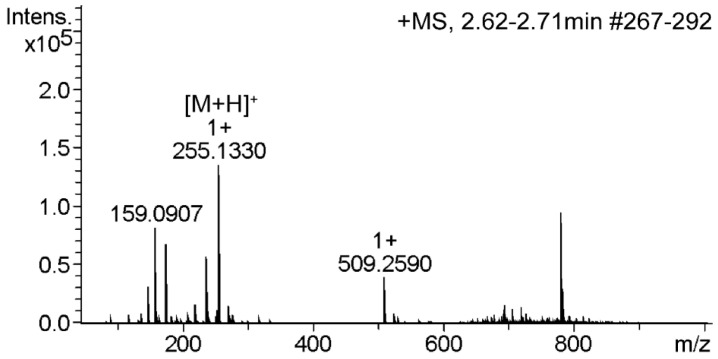
Product ion scan of pyrraline in a PED sample recorded by UPLC-UV-MS/MS (parent ion, *m*/*z* 255.1330).

In the present study, all quantitative data were obtained by ultrahigh-performance liquid chromatography with UV-visible detector coupled with tandem mass spectrometry (UPLC-UV-MS). The concentrations of pyrraline were determined based on the chromatograms measured in the selected ion recording (SIR) mode at *m*/*z* 255. Although the validation of SPE procedure was also monitored by UPLC-UV-MS, the validation results were presented by ultrahigh-performance liquid chromatography with UV-visible (UPLC-UV) chromatogram. Since the mass detector with SIR mode was used for quantification, it was unscientific to validate the SPE procedure using mass detector. In order to test the effect of matrix components in different PEDs on the UPLC separation and UV detection of pyrraline, several PEDs were analyzed without the SPE application. In these experiments, many signals were detected (Figure 5a). Although pyrraline could be separated from other contaminants on the chromatogram, those signals showed a low response. Thus, reliable quantification of pyrraline in PEDs required sample purification before UPLC analysis. The contaminants could be effectively separated from pyrraline using the optimized SPE protocol; meanwhile, a high peak response of pyrraline could be obtained (Figure 5b). The MS spectrum of pyrraline is presented in Figure 6.

### 2.4. Validation of the Method

Prior to quantification, the linearity of the calibration, repeatability, recovery and limit of detection (LOD)/limit of quantification (LOQ) were determined. Results of the calibration for pyrraline (0.1–50.6 μg/mL) were as follows: 0.0921*x* − 0.0120, *R*^2^ = 0.99990. The introduction of optimized SPE into the UPLC-UV-MS detection gained an excellent LOD (30.4 ng/mL) and LOQ (70.3 ng/mL) in PEDs. In addition, the application of optimized SPE led to an excellent removal of contaminants, while simultaneously obtaining good recoveries of pyrraline ranging between 93.2% and 98.5% and a high reproducibility with relative standard deviations (RSD) of <5% (Table 1). Therefore, the optimized SPE procedure before UPLC-UV-MS analysis allowed reliable quantification of pyrraline in PEDs.

### 2.5. Quantification of Free-Pyr and Pep-Pyr in Commercial PEDs

After validation, the method was applied to determine Free-Pyr and Pep-Pyr in 27 different PED samples (Table 2). Nine of the PED samples were enriched with WPH (Group 1), nine with SPH (Group 2), and nine with CPH (Group 3). For Free-Pyr in PEDs, the amount of Free-Pyr in Group 1 was below 7.75 mg/100 g of protein, Group 2 contained <3.05 mg/100 g of protein. In Group 3, the amount of Free-Pyr was below 3.05 mg/100 g of protein.

The amount of Pep-Pyr in Group 1 ranged between 27.03 and 76.05 mg/100 g of protein (median = 54.08 mg/100 g of protein), whereas Group 2 contained 15.35–39.03 mg/100 g of protein (median = 31.00 mg/100 g of protein). In Group 3, amounts of Pep-Pyr ranged between 22.55 and 46.98 mg/100 g of protein (median = 33.73 mg/100 g of protein).

**Figure 7:**
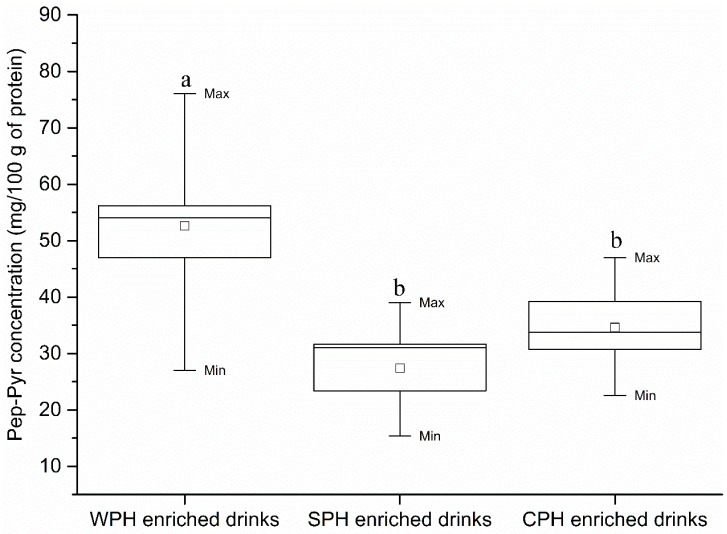
Box plot with whiskers from minimum to maximum of Pep-Pyr concentration in different groups of peptides enriched drinks clustered to the type of peptides source. □ is the mean value. Different characters (a, b) on boxes indicate significant difference between groups (*p* < 0.05).

In Figure 7, significant differences in Pep-Pyr concentration were shown between Groups 1 and 2 as well as between Groups 1 and 3. In addition, no significant difference in Pep-Pyr content between Groups 2 and 3 was observed. Consequently, a conclusion can be drawn that the concentration of Pep-Pyr in WPH-enriched drinks was higher than that in CPH- and SPH-enriched drinks.

In terms of total pyrraline content, a similar result was observed as compared with Pep-Pyr content. In summary, in all PEDs, the most abundant pyrraline was in the form of Pep-Pyr (accounting for above 96 percent of total pyrraline), Free-Pyr was present in a small proportion of total pyrraline (less than four percent of total pyrraline).

## 3. Discussion

During food processing, pyrraline can be formed by reaction between the reducing saccharides and amino groups. In previous studies, total amount of pyrraline has been frequently determined in different commercially available foods, such as milk products, bakery products and commercial processed carrot samples [35,36,37]. Advantages of protein hydrolysates include its large amount of di- and tri-peptides (other than intact proteins and free amino acids) for skeletal muscle protein anabolism and short-chain peptides for perceived health effects [24,38]. Consequently, protein hydrolysate has been frequently used in commercial drinks for nutrition recovery and health function, and these beverages may be an important extrinsic source of pyrraline, especially the Pep-Pyr.

In the present study, Free-Pyr and Pep-Pyr were quantified individually for the first time in commercial PEDs. For pyrraline analysis in PEDs, the interfering matrix components may significantly restrict the application of UPLC-UV-MS method without SPE, and thus purification of pyrraline from the sample matrix by SPE prior to UPLC-UV-MS analysis was crucially required. After optimization of SPE protocol, significant removal of interferents, high recovery rates (ranging between 93.2% and 98.5%) and high reproducibility (RSD of <5%) were obtained.

Ten microliter of OPD solution with 0.4 mg/mL were added to 90 μL commercial PEDs, which could lead to a complete deactivation of 1,2-dicarbonyl compounds. No significant difference in 3-DG content among different kinds of PEDs (Figure 3) was found. This result may be attributed to the insignificant difference in reducing sugar content among types of PEDs (as shown in Table 3). Aside from being the predominant 1,2-dicarbonyl compound in commonly consumed foods, 3-DG were also found to be primarily derived from degradation of reducing carbohydrates [30,39,40,41,42]. As a result, the 3-DG content is closely related to reducing sugar content in PEDs.

In terms of pyrraline content in commercial food products, Chiang [19] reported that 1.96–133 mg/kg of pyrraline can be detected during the heating of nonfat dry milk heated at 80 °C for 0–6 h. Up to 40 mg of protein-bound pyrraline/100 g of protein was found by Resmini [36] in commercial spaghetti when dry temperatures above 75 °C. Henares [43] reported that 43.2–49.5 mg/100 g protein and 26.5–49.5 mg/100 g protein of pyrraline were detected in two types of enteral formula with same components but different protein content. Wellner [37] found up to 134 mg/kg protein of pyrraline in processed carrot juices. Comparing with above literature data, it seems that the amount of total pyrraline in WPH-enriched drinks (56.10 mg/100 g protein) is quite high. In addition, the content of total pyrraline in SPH- (16.23–39.03 mg/100 g protein) and CPH-enriched drinks (28.70–46.98 mg/100 g protein) are also close to that in commercial spaghetti (up to 40 mg/100 g protein) and enteral formula samples (26.5–49.5 mg/100 g protein), respectively.

For the comparison between PEDs and other food products (nonfat dry milk, commercial spaghetti, enteral formula and processed carrot juices), the difference in pyrraline content is attributed to the difference of food ingredients, especially the existence forms of amino nitrogen. In PEDs, the majority of amino nitrogen was derived from short chain peptides (Table 3) as di- to penta-peptides (accounting for above 70% of peptides in PEDs), which was consistent with the manufacturer’s claims. However, instead of peptide, protein was the major form of amino nitrogen in other food products in the previous studies. Pyrraline is an AGE derived from the reaction between carbonyl group of reducing saccharides and the ε-NH_2_ of lysine residues. The ε-NH_2_ modification of lysine residue may occur in lower frequency in proteins than that in peptides due to the three-dimensional structures of proteins and can be hindered in proteins. Besides, the reactivity of peptides below 1000 Da is higher than that of peptides with higher molecular weight in Maillard reaction [44]. As shown in Table 3, di- to penta-peptides was the most abundant amino nitrogen presented in PEDs. As a result, the pyrraline content in PEDs was generally higher than that in other food products form previous studies. Consequently, as implied in the present study, both advantages and disadvantages of protein hydrolysates caused by food processing should be taken into consideration for application of protein hydrolysates to commercial food products.

The Free-Pyr was a negligible portion (less than 4%) of the total pyrraline, and no obvious trend in Free-Pyr content was observed among WPH-, SPH- and CPH-enriched drinks. The existence of Free-Pyr in PEDs may be due to the high degree of hydrolysis of protein hydrolysates and the storage conditions of PEDs. As shown in Table 3, a tiny portion of free amino acid (3.2%–4.8% of amino nitrogen), including lysine, can be released from protein during the protein hydrolysates’ production, indicating only release of free lysine (such as Free-Pyr) in small amount from hydrolysis of whey protein, soy protein and collagen.

PED was previously proved to be an important extrinsic source of Pep-Pyr, and Pep-Pyr was also found to be the major existence form of pyrraline in all PEDs in the present study. The concentration of Pep-Pyr in drinks enriched with WPH was higher than that with SPH or CPH. For the pyrraline formation, lysine is the most frequently affected amino acid, since its amino group of side chain continues to be potentially available when bound into peptides and proteins [45]. In summary, two factors that may contribute to the difference in Pep-Pyr content among these three types of PEDs include the distribution of peptide lengths in PEDs and the amino acid compositions of protein. For the former, Lan et al. [46] have found that peptides below 1000 Da abundant in protein hydrolysates decreased more rapidly than peptides between 1000 and 5000 Da and peptides above 5000 Da with increasing temperatures in the Maillard reaction, implying that the reactivity of low molecular weight (below 1000 Da) peptides is higher than that of high molecular weight (above 1000 Da) peptides. De Kok and Rosing [47] have shown that the relative reactivity of peptides and amino acid was Gly–Gly > Gly–Gly–Gly >> Gly in the glucose-glycine homopolymers model systems, the reactivity of peptides was much greater than that of the free amino acids.

From the summary table describing the amino acid compositions of different commercial protein sources (Table 4) [27], the approximate concentrations of lysine residue were 10.2 g/100 g, 6.3 g/100 g and 4.1 g/100 g in whey protein, soy protein and collagen, respectively. Since lysine residue is an important nitrogen source of pyrraline, various concentrations, in addition to the reactivity of individual lysine residue in protein, have contributed to the difference in Pep-Pyr content in PEDs. Therefore, PEDs containing higher content of lysine residue can have higher probability of pyrraline formation.

Consequently, when comparing pyrraline content among these three different types of PEDs, both the distribution of peptide lengths and the amino acid compositions of protein should be systematically taken into consideration. Compared to the two other types of PEDs, WPH-enriched drinks contained high proportion of di- to penta-peptides and high content of lysine residue, which may lead to high concentration of pyrraline. Although the content of lysine residue in SPH-enriched drinks was higher than that in CPH-enriched drinks, an opposite result in proportion of di- to penta-peptides was obtained, which may explanation why no significant difference in pyrraline content was observed between SPH- and CPH-enriched drinks.

Actually, the formation of pyrraline may be also attributed to the reducing sugar content and the structure of peptides, especially the amino acid adjacent to lysine. The nature of the vicinal amino acids strongly affects lysine reactivity towards saccharides. The lysine reactivity can be strongly improved in the presence of hydrophobic residues (such as Ile, Leu and Phe) adjacent to lysine [48]. However, both the adjacency of these hydrophobic amino acids (e.g., Ile, Leu and Phe) to lysine and its effect on Pep-Pyr formation still remain unclear. Investigation on the effect of peptide sequences in detail in peptide-glucose model systems is required in future study.

## 4. Materials and Methods

### 4.1. Chemicals

All chemicals used were of analytical grade unless otherwise stated. Aminopeptidase M was obtained from Merck (Darmstadt, Hesse, Germany). Pepsin, pronase E and prolidase were purchased from Sigma-Aldrich (Shanghai, China). Acetonitrile and formic acid were HPLC grade from Merck (Darmstadt, Hesse, Germany). The solid-phase extraction (SPE) cartridge, Cleanert PEP-2 SPE cartridge (200 mg/6 mL, Bonna-Agela Technologies Inc., Tianjin, China), was used for purification of Maillard reaction products. 3-Deoxyglucosone (3-DG, purity > 99.99%) was obtained from Toronto Research Chemicals (Toronto, ON, Canada). Pyrraline standard sample (purity > 99.99%) was purchased from PolyPeptide Laboratories (San Diego, CA, USA).

### 4.2. PED Samples

In this study, 27 different PED samples (ingredients: vitamin, sugar, glucose, protein hydrolysates, caffeine, etc.) were purchased from local stores. Nine of the PED samples were enriched with WPH (Group 1), Nine with SPH (Group 2), and nine with CPH (Group 3). Moreover, an energy drink sample (ingredients: vitamin, sugar, glucose, caffeine, etc.) was chosen as a pyrraline-free matrix (PFM) due to absence of amino acid residue groups. In addition, a sugar-free drink (ingredients: protein hydrolysates, amino acids, aspartame, vitamin, etc.) was purchased as a 3-DG free matrix. Aliquots of 10 mL were taken from all samples and degassed for 15 min by sonication.

### 4.3. Protein Content

Protein determination was carried out by the Kjeldahl method [49].

### 4.4. Peptide Lengths Measurement

The distribution of peptide chain lengths in PEDs was obtained by using an adapted automated Edman degradation [50]. Values for Cys and Trp were not taken into account. Briefly, the peptide sequence was obtained by using a gas-liquid solid phase peptide and protein sequenator with 16 cycles in the sequenator program algorithms [51]. The amino acids were analyzed by automated precolumn derivatization and high-performance liquid chromatography [52]. The large peptides (>1.5 kDa) were determined by a tricine-sodium dodecyl sulfate-polyacrylamide gel electrophoresis (tricine SDS-PAGE) procedure [53].

### 4.5. Total Reducing Sugar Content Measurement

The total reducing sugar content in PEDs was determined by the 3,5-dinitrosalicylic acid colorimetry (DNS) method [54].

### 4.6. Optimization of Derivatization Procedure

In order to terminate the Maillard reaction, 1,2-dicarbonyl compounds in PEDs were derivatizated as quinoxaline by reaction with *o*-phenylenediamine (OPD) (Figure 1). The derivatization of 1,2-dicarbonyl compounds in PEDs was carried out with OPD in a 4-(2-hydroxyethyl)-1-piperazineethanesulfonic acid buffer (HEPES, 0.1 M, pH 6.8), typically OPD concentrations of 0.1–0.8 mg/mL were used. Ten microliters of OPD solution was added to 90 μL of the PED samples. The samples were mixed immediately and kept in the dark overnight, then filtered by nylon membrane (0.45 μm) before SPE procedure and chromatographic analysis.

### 4.7. Optimization of SPE Procedure

The cartridge for pretreatment was used as follows (flow rate: 1.0 mL/min):

(a) The cartridge was preconditioned with 4 mL of methanol, and equilibrated with 4 mL of water before loading the sample.
(b) One milliliter of sample was then applied to the cartridge, followed by washing the cartridge with 1 to 7 mL of water.
(c) Finally, the target compounds were eluted from the cartridge with 1 to 7 mL of acetonitrile, and the eluent was evaporated to dryness at 50 °C by pressure blowing concentrator.
(d) The dried residue was then dissolved in 1 mL of HPLC eluent: 0.1% formic acid in water containing 15% (*v*/*v*) acetonitrile.

The SPE pretreated samples were filtered by membrane (0.45 μm) before qualitative detection.

### 4.8. Quantification of Free-Pyr and Pep-Pyr

After the SPE procedure, the samples were subjected to the *ultrahigh-performance liquid chromatography* system with UV-visible detector coupled with tandem mass spectrometry (UPLC-UV-MS). The content of pyrraline was employed as the Free-Pyr content in PED samples.

For total amount of pyrraline in PED samples, samples without SPE pretreated were subjected to a complete enzymatic hydrolysis procedure [55]. After the complete hydrolysis procedure, the supernate was collected after centrifugation (10,000 rpm, 25 min), and then was subject to SPE procedure as described previously. After SPE, the samples were subjected to UPLC-UV-MS analysis. The content of pyrraline was employed to quantify the total content of pyrraline in PED samples.

Consequently, the content of Pep-Pyr was calculated as follows: content of Pep-Pyr = total content of pyrraline − content of Free-Pyr.

### 4.9. Quantification of 3-DG

The same optimized SPE procedure was also applied to 3-DG_qx_ purification. After the SPE procedure, the samples were subjected to the UPLC-UV-MS.

### 4.10. UPLC-UV-MS

A 10-μL aliquot of the derivatized and purified sample was assayed by an UPLC-UV-Q-TOF system. The system was an Agilent 1290 system (Agilent Technologies, Inc., Palo Alto, CA, USA) coupled to Bruker microTOF-q II mass spectrometer (Bruker Corporation, Bremen, Germany). The chromatographic column was an Agilent ZORBAX SB-C18 column (2.1 mm × 150 mm, 5 μm), and the temperature was set to 30 °C. The injection volume was 5 µL, mobile phase solvents consisted of 0.1% formic acid in water (A) and 0.1% formic acid in acetonitrile (B). The gradient conditions were 5% B (0 min), 5% B (0.8 min), 40% B (8 min), 5% B (10 min), and 5% B (12 min). The flow rate was 0.2 mL/min.

Signals were monitored with UV-visible detector at 298 nm for pyrraline and at 316 nm for 3-DG_qx_. For the MS/MS experiments, the ESI source conditions were as follows: endplate off, −500 V; capillary voltage, 4.5 kV; nebulizer pressure, 0.3 bar; dry gas flow, 4.0 L/min; and dry temperature, 180 °C. Mass scan range was 50 to 1000 *m*/*z* in positive mode. Sum formula generation was processed by SmartFormula 3D and the FragmentExplorer (Bruker Daltonics GmbH, Bremen, Germany), and fragment structures were assigned by Data Analysis 4.1 (Bruker Daltonics GmbH). Profile Analysis 2.1 (Bruker Daltonics GmbH) was used for statistical data evaluation. Pyrraline was quantified by its parent ion peak (*m*/*z* = 255.1300), and 3-DG was determined by the parent ion peak of 3-DG_qx_ (*m*/*z* = 235.1040). Selected ion recording (SIR) mode was used for quantification. External calibration was performed with the standard. External calibration of pyrraline with pyrraline-free matrix involved was prepared at different analyte concentrations and linear calibrations calculated for each analyte. The same method was employed in external calibration of 3-DG with 3-DG free matrix involved instead of pyrraline-free matrix.

### 4.11. Validation of the Method and Quantification of Pyrraline

In order to determine the concentration of pyrraline in PED samples, a calibration curve was prepared by pyrraline standard which covered the expected concentration range: a nine-point calibration curve was used for pyrraline. Linearity of each calibration curve was tested by linear regression analysis with a minimally acceptable correlation coefficient of 0.9990.

Repeatability was calculated from three independent measurements and was performed as RSD. LOD and LOQ were determined by the signal-to-noise ratio (S/N). Water (*n* = 10) was used to define the noise, in which LOD was calculated as S/N 3:1 and LOQ as S/N 10:1.

To evaluate the matrix influence, the recovery rate of pyrraline was determined by analyzing spiked pyrraline in the PFM after SPE application. For each concentration level of standard pyrraline, the recovery rate was calculated as (spiked pyrraline concentration/added pyrraline concentration) × 100%.

## 5. Conclusions

Compared to the literature data, the total pyrraline content was quite high because the short chain peptides were the main form of amino nitrogen in PEDs. Free-Pyr was a small proportion of total pyrraline. Pep-Pyr was identified as the major form of pyrraline in PEDs. Formation of Pep-Pyr from PEDs was dependent on the class of protein hydrolysate source. The difference in Pep-Pyr content among WPH-, SPH- and CPH-enriched drinks was attributed to the distribution of peptide lengths and the amino acid compositions of protein in PEDs. PEDs with higher distribution of di- to penta-peptides and higher content of lysine residue can produce greater amounts of pyrraline. Therefore, when we are enjoying the nutritional advantages of protein hydrolysates applied to commercial food products, the potential risk of Pep-Pyr derived from protein hydrolysates caused by food processing cannot be ignored.

## Figures and Tables

**Table 1:** Repeatability and recovery rates of different concentrations of pyrraline and 3-DG.

			RSD ^a^		RSD ^a^		RSD ^a^		RSD ^a^
pyrraline	μg/mL ^b^	63.5		34.6		10.0		0.5	
	Recovery	98.5%	2.5%	95.4%	2.3%	94.1%	4.8%	93.2%	3.4%
3-DG	μg/mL ^c^	48.6		26.3		10.0		0.5	
	Recovery	99.2%	5.6%	96.3%	6.5%	95.6%	5.3%	94.7%	6.3%

^a^ Relative standard deviations (RSD); ^b^ Concentration of pyrraline in pyrraline-free matrix (PFM); ^c^ Concentration of 3-DG in 3-DG free matrix.

**Table 2:** Concentrations of free-form and peptide bound pyrraline in 27 commercial PEDs.

Sample	Peptide Source	Free-Form Pyrraline (mg/100 g of Protein)	Peptide Bound Pyrraline (mg/100 g Protein)	Total Pyrraline (mg/100 g of Protein)
Group 1				
PED1	WPH	nd	45.23 ± 1.95	45.23 ± 1.95
PED2	WPH	5.60 ± 1.15	76.05 ± 0.60	81.70 ± 0.38
PED3	WPH	tr	52.65 ± 2.30	52.65 ± 2.30
PED4	WPH	nd	59.15 ± 1.58	59.15 ± 1.58
PED5	WPH	tr	56.10 ± 0.30	56.10 ± 0.30
PED6	WPH	7.75 ± 0.75	27.03 ± 0.58	34.78 ± 1.33
PED7	WPH	nd	56.18 ± 0.35	56.18 ± 0.35
PED8	WPH	3.25 ± 0.23	46.95 ± 0.55	50.20 ± 2.55
PED9	WPH	2.95 ± 0.05	54.08 ± 0.40	57.08 ± 0.83
Median			54.08	56.10
Average Percentage		3.99%	96.01%	100%
Group 2				
PED10	SPH	tr	39.03 ± 1.80	39.03 ± 1.80
PED11	SPH	nd	16.23 ± 1.80	16.23 ± 1.80
PED12	SPH	tr	31.63 ± 1.98	31.63 ± 1.98
PED13	SPH	3.00 ± 0.20	15.35 ± 0.15	18.35 ± 1.10
PED14	SPH	3.05 ± 0.30	23.40 ± 0.53	26.53 ± 1.53
PED15	SPH	nd	31.00 ± 1.28	31.00 ± 1.28
PED16	SPH	nd	31.43 ± 1.70	31.43 ± 1.70
PED17	SPH	2.53 ± 0.10	27.08 ± 0.08	29.60 ± 2.58
PED18	SPH	tr	31.65 ± 0.60	31.65 ± 0.60
Median			31.00	31.00
Average Percentage		3.39%	96.61%	100%
Group 3				
PED19	CPH	3.35 ± 0.80	31.18 ± 0.60	34.58 ± 1.78
PED20	CPH	tr	30.70 ± 1.00	30.70 ± 1.00
PED21	CPH	nd	33.73 ± 1.53	33.73 ± 1.53
PED22	CPH	nd	35.38 ± 1.30	35.38 ± 1.30
PED23	CPH	nd	29.65 ± 1.30	29.65 ± 1.30
PED24	CPH	tr	46.98 ± 1.73	46.98 ± 1.73
PED25	CPH	6.13 ± 0.28	22.55 ± 0.30	28.70 ± 0.73
PED26	CPH	nd	39.23 ± 2.05	39.23 ± 2.05
PED27	CPH	nd	42.18 ± 2.65	42.18 ± 2.65
Median			33.73	34.58
Average Percentage		2.98%	97.02%	100%

(Value ± standard deviation; *n* = 3); nd, not detected; tr, trace amounts (between LOD and LOQ).

**Table 3:** Protein content, total reducing sugar content and peptide lengths distribution in commercial PED samples.

	Group 1 (WPH)	Group 2 (SPH)	Group3 (CPH)
Protein content (g/100 mL)	4.0 ± 0.3 ^a^	3.6 ± 0.6 ^a^	3.9 ± 0.2 ^a^
Total reducing sugar content (g/1000 mL)	82.2 ± 5.0 ^a^	85.6 ± 3.6 ^a^	84.5 ± 6.3 ^a^
Distribution of peptide lengths (%)			
1 (free amino acids)	4.2 ± 0.3 ^a^	3.2 ± 1.1 ^b^	4.8 ± 1.4 ^a^
2–5	80.3 ± 1.0 ^a^	70.0 ± 2.0 ^b^	76.3 ± 1.5 ^c^
6–10	8.2 ± 1.2 ^a^	12.4 ± 1.3 ^b^	9.0 ± 0.8 ^a^
11–15	5.1 ± 0.9 ^a^	7.3 ± 5.8 ^a^	6.7 ± 0.2 ^a^

^a–c^ Means ± standard deviation with different superscripts within same row indicate significant difference (*p* < 0.05).

**Table 4:** Approximate essential amino acid profile of various protein sources [27].

Essential Amino Acid	Whey Protein	Soy Protein	Collagen Protein
Ile	5.5	4.9	1.7
Leu	14.2	8.2	3.4
Lys	10.2	6.3	4.1
Met	2.4	1.3	0.5
Phe	3.8	5.2	2.0
Thr	5.5	3.8	2.1
Trp	2.3	1.3	na
Val	5.9	5.0	3.0
Total	42.7	36.0	16.8

Approximate concentration of essential amino acids present within various forms of commercially available protein (g/100 g). na, indicates data not available.
